# A Retrospective Cohort Study on Coronary Endarterectomy Outcomes in Coronary Artery Bypass Graft Patients

**DOI:** 10.7759/cureus.4279

**Published:** 2019-03-19

**Authors:** Hafez Mohammad Abdullah, Adnan Khan, Momin Khan Afridi, Azam Jan, Uzma I Khan, Waqas Ullah, Asrar Ahmad, Muhammad Omar, Ishaq Khan, Arsalan Khan

**Affiliations:** 1 Internal Medicine, University of South Dakota Sanford School of Medicine, Sioux Falls, USA; 2 Cardiology, Hayatabad Medical Complex, Peshawar, PAK; 3 Cardiology, Rehman Medical Institute, Peshawar, PAK; 4 Cardiothoracic Surgery, Rehman Medical Institute, Peshawar, PAK; 5 Internal Medicine, Khyber Teaching Hospital, Peshawar, PAK; 6 Internal Medicine, Abington Hospital-Jefferson Health, Abington, USA

**Keywords:** revascularization, coronary endarterectomy, coronary artery bypass graft (cabg)

## Abstract

Introduction: The aim of this study is to determine the outcomes following coronary endarterectomy (CE) in patients who underwent coronary artery bypass grafting (CABG) for revascularization in our hospital.

Methods: We retrospectively reviewed patients who underwent CABG over a six-month period, from November 1, 2016 to May 31, 2017 and examined their outcomes in regards to CE.

Results: A total of (n=361) CABG procedures were performed in our study period, though complete records were available for only (n=254) patients. Amongst these, (n=37) patients (14.5%) required CE. Ages ranged from 43 to 75 years for these patients, (n=7) were females and (n=30) males. Comorbidities included hypertension in (n=19) patients, diabetes in (n=12) patients and hepatitis B in (n=11) patients. The right coronary artery (RCA) was the most common artery endarterectomized (n= 15), followed by the left anterior descending (LAD) (n= 10), obtuse marginal (n= 6 patients), diagonals (n=5) and ramus (n=2). Two vessels were endarterectomized in (n=4) patients. A total of (n=9) patients had two-vessel CABG, (n=16) had three-vessel CABG and (n=8) had four-vessel CABG. The left internal mammary artery (LIMA) was used in (n=25) patients. Two patients required intra-aortic balloon pump post-operatively. All the patients had received inotropic support postoperatively in the intensive care unit (ICU). There were no reports of postoperative mortality. One patient remained in the ICU for four days postoperatively, the rest of the patients were stepped down to the ward in less than four days.

Conclusions: CE is a safe and viable option as an adjunct to CABG in long segment totally occluded vessels needing revascularization and reconstruction.

## Introduction

Coronary endarterectomy (CE) is a useful adjunct procedure generally done in the presence of diffuse coronary artery disease. It aims to restore uninterrupted blood flow by excising diseased segments as well as atheromatous deposits. It is a procedure necessitated in complicated cases such as diffuse coronary artery occlusion. CE is mostly done in patients where there is a long segment total occlusion of a dominant vessel and no available place to appropriately anastomose. CE was initially developed in the 1950s and was performed on patients with severe three-vessel disease [1]. Although it has proven to be an effective collateral technique, there is evidence to suggest that it has been associated with significant postoperative morbidity and mortality and fell out of favor for a while [2-3]. However, with improvement in surgical techniques, more recent studies have demonstrated the efficacy and safety of CE in carefully selected cohorts [4-7]. For example, a retrospective study showed that in some instances, long arteriotomy in combination with the reconstruction of the left anterior descending (LAD) artery is required for the excision of an atherosclerotic plaque, with favorable early and late outcomes, thus advocating CE as a safe option for diffuse coronary artery disease [8].

With the increasing incidence of severe three-vessel coronary artery disease, it is predicted that it would be increasingly used in the future. Soylu et al. conducted a meta-analysis at the Imperial College London and found that the profile for patients undergoing CABG was made up of a growing proportion of patients who were older, had more diffuse coronary disease, had many comorbidities, and who may have also undergone previous percutaneous coronary interventions (PCIs) [9]. The study also looked into intensive therapy unit (ITU) stay, hospital stay, and long-term graft failure [9]. We have also noticed an increasing incidence in the number of cases undergoing endarterectomy in our institution. The cohort of patients referred to our institution belongs to a demographic which includes older patients with more severe coronary artery disease.

Soylu et al., in their meta-analysis, also evaluated the technique (either open or closed) and found it to be among variables determining perioperative and postoperative mortality [9]. High cross-clamp times and bypass times have been identified as independent risk factors and predictors of mortality in patients who were selected for CE. In both low and high-risk cardiac patients, higher cross-clamp times have been correlated with increased hospital mortality, longer hospitalization, mechanical ventilation, greater requirements for blood transfusion, and renal complications. Aortic cross-clamp time is an index of myocardial ischemia and is associated with worse prognosis in high-risk cardiac surgery [10]. However, in another independent study, CE was associated with prolonged clamp and bypass times as well as ventilator times in patients as compared to patients who had CABG alone; postoperative mortality and complications were comparable, although they incurred longer intensive care unit (ICU) and hospital stays. The report concluded that CE was a viable and safe option as an adjunct for revascularization [11].

Off-pump coronary artery bypass grafting (OPCABG) is another method of revascularization in diffuse coronary artery disease. A study that looked at the safety of off-pump coronary artery bypass with CE (OPCAB-CE) vs. on-pump coronary artery bypass with CE (ONCAB-CE) showed comparable 30-day mortality. This was based on a study reviewing 71 articles of which nine were eventually analyzed. A conclusion was drawn that (OPCAB-CE) was a safe and feasible method with equivalent results as compared to (ONCAB-CE) [12]. Other studies also support this finding [13].

Generally, cardiac surgeons prefer arterial grafts over venous in CABG procedures as arterial grafts are better suited to manage blood pressure in the coronary vessels, which is why the left internal mammary artery (LIMA) is the most commonly used arterial graft, frequently anastomosed to the LAD. LIMA patency rates are 90% in a ten-year period and, in combination with extensive LAD endarterectomy and reconstruction, it may be a safe and feasible option for diffuse arterial disease [14].

## Materials and methods

This retrospective cohort study was conducted between November 1, 2016 to May 31, 2017, a six-month period during which (n=362) patients underwent CABG; complete records for only (n=254) patients were found. All preoperative data, including patient demographics, preoperative medications, were obtained from the records section of Rehman Medical Institute’s Cardiac Surgery database after approval from our Institutional Review Board. Data were collected on all patients who required CE during the surgery. We analyzed the outcomes of our patients and attempted to identify any variable affecting the outcomes.

## Results

During the study duration, the total bypass graft procedures performed were (n=362) and we were able to get complete records of (n=254) patients. On review, we found that a total of 37 or 14.5% of the patients undergoing CABG had undergone CE too. The age range at the time of surgery of these patients was 43 to 75 years with the average being 58.92. The total number of men were (n=30) or (81.08%) and (n=7) or (18.92%) were females (Table 1).

**Table 1:** General patient characteristics BMI-body mass index.

Demographics	
Age	Mean 59.92 years (Range 43-75)
BMI	Mean 28.26 (Range 17.6-43.6)
Weight	Mean 77.22 kg (Range 53-105)
Gender	Males 81.08%, Female 18.92%
Nationality	Pakistani 34, Afghani 03

An analysis was done on the comorbidities among the sample population of which (n=19) patients had hypertension, (n=12) were diabetic, and one patient had hepatitis B (Table 2). We found that a majority of the patients were taking preoperative statins, nitrates, beta blockers, and antiplatelets.

**Table 2:** This table depicts patient co-morbidities and pre-op medications HTN-hypertension; DMII-type 2 diabetes; IHD-ischemic heart disease.

Co-morbidities	Pre-op medications
HTN 51.35%	Statins 40.54%
DMII 29.73%	Anti-platelets 37.84%
IHD 10.81%	Nitrates 32.43 %
Thyroid pathology 2.70%	Beta Blockers 29.73%
Hepatitis B 2.70%	Oral hypoglycemic 21.62%

In our analysis, the most common artery endarterectomized was the right coronary artery (RCA) in (n=15) patients, followed by the LAD in (n=10) patients, obtuse marginal (OM) in (n=5) patients (n=2 in OM1, n=1 in OM2, n=2 in OM3), ramus in (n=2) patients, diagonal in (n=4) patients (n=1 in high diagonal and n=3 in diagonal 1), and n=1 in the posterior descending artery (PDA) (Table 3).

**Table 3:** This table depicts the frequency of the various coronary arteries endarterectomized

Coronary artery	Number of patients (total n=37)
Right coronary artery	15
Left anterior descending	10
Obtuse marginal	5
-Obtuse marginal 1	2
-Obtuse marginal 2	1
- Obtuse marginal 3	2
Ramus	2
Diagonal	4
-High diagonal	1
-Diagonal 1	3
Posterior descending artery	1

We also looked into patients who had more than one vessel endarterectomized, with (n=4) patients having two vessels endarterectomized. Also, (n=9) patients had two-vessel CABG, (n=16) patients had three-vessel CABG, and (n=8) had four-vessel CABG.

**Figure 1:**
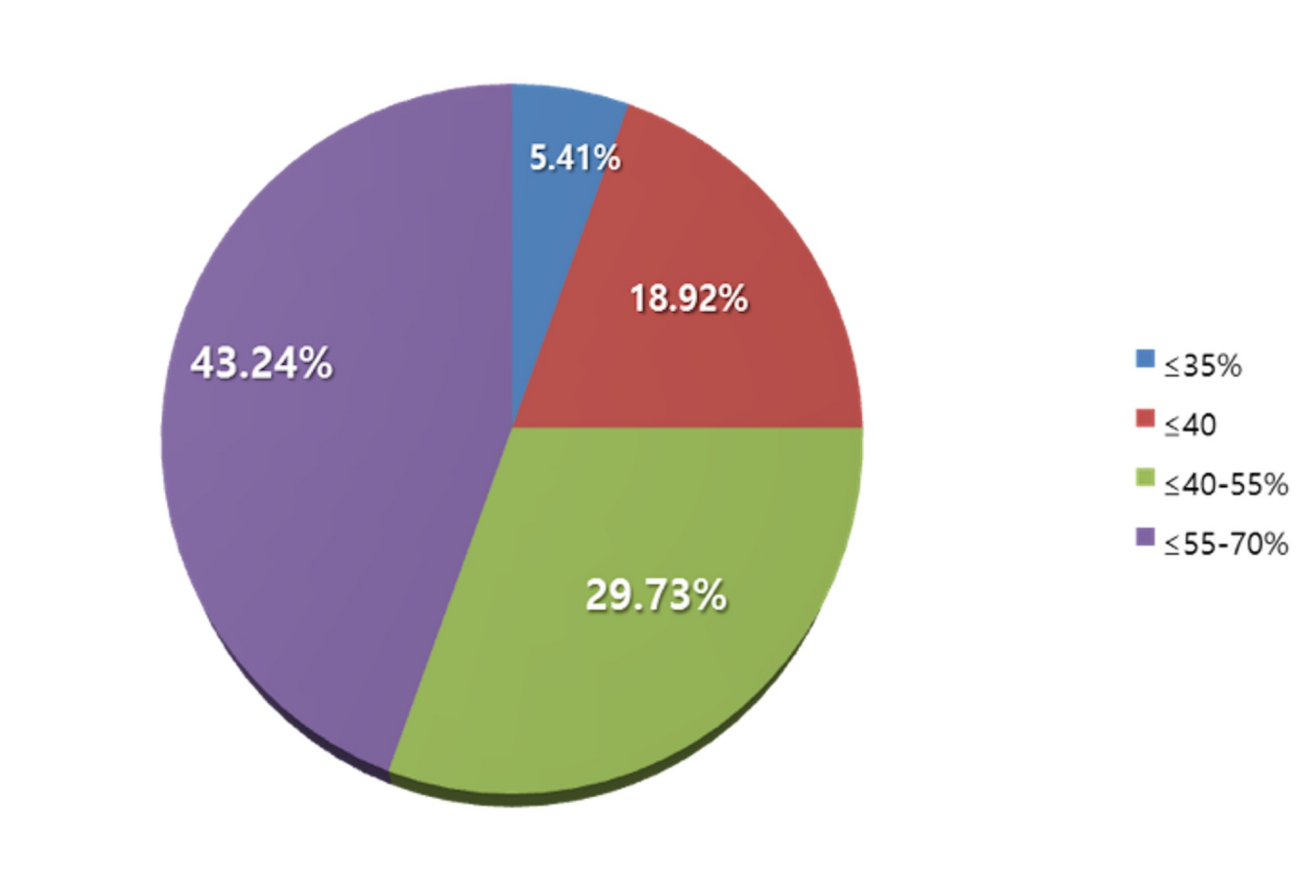
This chart depicts the preoperative left ventricular ejection fraction of our patient cohort

About 43% of the patients had an ejection fraction in the range of ≤55%-70%, 29% of them being between ≤40%-55%, with 18% having an ejection fraction of ≤40%; for just two patients, the ejection fraction was ≤=35% (Figure 1).

LIMA was grafted in 25 patients, 4 patients had required an intra aortic balloon pump (IABP), and all patients received post-operative inotropes used in the ICU. Average aortic clamp time was 54.80 minutes and cardiopulmonary bypass time 95.67 minutes. One patient had arrested on the table and one patient had to be reopened in the operation theatre. There was no incidence of postoperative respiratory failure and renal failure. A total of 18 (40.54%) patients out of 37 had packed red blood cell (PRBC) transfused while 12 (32.43%) patients had fresh frozen plasma (FFP) and platelets transfused. Average post-op stay in the hospital was 7.89 days with the exception of one patient who stayed for four days post operative in the ICU and the rest all remained ≤3 days. Three patients stayed for more than 10 days in the hospital. There was no post-op mortality in these patients. Table 4 depicts the postoperative characteristics of our cohorts.

**Table 4:** Outcomes in our patients ICU- Intensive care unit

Total n=37 patients	
Clamp time	Mean 54.80 minutes
Bypass Time	Mean 95.67 minutes
Red cell transfusion	40.54 %
Platelet transfusion	32.43 %
Fresh frozen plasma	32.43 %
Hospital stay	Mean 7.89 days
ICU stay	Mean 2.64 days
Ventilator times	Mean <24 hours
Dialysis	0 %
Intra Aortic Balloon Pump	10.81 %
Mortality	0 %

## Discussion

The incidence of three-vessel disease and diffuse coronary artery disease is increasing worldwide, necessitating the use of coronary artery endarterectomy as a procedure. The rate of CE with CABG surgeries is reported somewhere between 3.7%-42% at different institutions, which shows that a significant number of the population has required it. CE has recently been re-introduced into the mainstream once again, as an essential armament in a cardiac surgeon’s armory [4-6,15]. CE has a controversial history, as it was associated with high rates of mortality and morbidity in the 1950s when it was first introduced [1]. Despite the development of technology such as the bypass machine, as well as the availability of more sophisticated approaches in treating myocardial infarction (MI) such as percutaneous coronary intervention (PCI), the need for the complete revascularization of coronary vessels in cases of diffuse coronary artery disease is still being felt [16]. In cases like these, CE is seen as a viable means of effectively preserving functions through the removal of atherosclerotic plaque with viable myocardium. In the modern era of cardiac surgery, with pharmacological as well as technological advancements, it is possible to re-evaluate the role of CE as an adjunct procedure to CABG. The identification of the cardiac risk factors in this regard is important as the presence of comorbidities, particularly their expressions in patients with diffuse coronary artery disease, is significantly intense in comparison to the characteristics of the usual cohorts. Brenowitz described the risk factors for multiple endarterectomies. They include age >70 years, reoperation, insulin-dependent diabetes mellitus, female sex, and severe ventricular dysfunction [5]. The risk factors identified with mortality associated with CE were as follows: female gender, diabetes mellitus, left main disease, acute MI, previous myocardial revascularization and ejection fraction <35% [16]. From the operative perspective, an atherosclerotic plaque may be detached by CE either manually or by means of carbon dioxide, although the latter has been effectively abandoned, since it is particularly difficult in the left coronary artery, being small and having a multitude of branches. Although it is usually performed in a single manner, it has been cited to be performed in two, three, and up to seven vessels, with some authors associating the number of endarterectomies with the morbidity and mortality in these patients. CE requires a greater period of extracorporeal circulation and anoxia. The right coronary artery was the most common vessel chosen for an endarterectomy, due to it being technically feasible. Brenowitz introduced the concept of endarterectomy in the left coronary artery but was found responsible for immediate mortality in many patients [5]. The main cause of postoperative mortality has been reported to be acute MI due to thrombosis in the site of endarterectomy, but a significant reduction in mortality was reported in patient groups who were on antiplatelets and anticoagulants [16]. Christakis prospectively studied a cohort of 317 patients with endarterectomies and compared them with 911 patients who had a conventional bypass procedure done, finding no difference in the mortality between the compared groups [17].

We studied a set number of patients (n=254) within a specific time period of six months. A total of (n=37) of these patients underwent CE. There was no incidence of perioperative MI or death, as well as no incidence of postoperative MI or death, in the immediate period or in the 30 days following the procedure. Our cohort included a majority of patients (43.24%) who had good ejection fractions (55%-70%); 51% of our patients had hypertension, and 10.81% had prior PCI, 40.54% of our patients were on statins, and 37.84% were on antiplatelets. All the patients, on follow up, were inquired about their quality of life post procedure. All of them returned to class I functional status as defined by the New York Heart Association (NYHA).

## Conclusions

CE may be a safe and viable option, as an adjunct to CABG, in long segment totally occluded vessels needing revascularization and reconstruction. However, further larger studies are needed to elucidate its role as an adjunct to CABG, to identify high-risk patients, and to study its safety in different clinical settings.
